# A Comparison between Porous to Fully Dense Electrodeposited CuNi Films: Insights on Electrochemical Performance

**DOI:** 10.3390/nano13030491

**Published:** 2023-01-25

**Authors:** Xuejiao Wang, Jingyuan Bai, Meilin Zhang, Yuxi Chen, Longyi Fan, Zhou Yang, Jin Zhang, Renguo Guan

**Affiliations:** 1 School of Materials Science and Engineering, Northeastern University, Shenyang 110819, China; 2 Key Laboratory of Near-Net Forming of Light Metals of Liaoning Province, Dalian Jiaotong University, Dalian 116028, China; 3 Engineering Research Center of Continuous Extrusion, Ministry of Education, Dalian Jiaotong University, Dalian 116028, China

**Keywords:** CuNi system, electrodeposition, porous film, fully dense film, hydrogen evolution reaction

## Abstract

Nanostructuring of metals is nowadays considered as a promising strategy towards the development of materials with enhanced electrochemical performance. Porous and fully dense CuNi films were electrodeposited on a Cu plate by electrodeposition in view of their application as electrocatalytic materials for the hydrogen evolution reaction (HER). Porous CuNi film were synthesized using the hydrogen bubble template electrodeposition method in an acidic electrolyte, while fully dense CuNi were electrodeposited from a citrate-sulphate bath with the addition of saccharine as a grain refiner. The prepared films were characterized chemically and morphologically by scanning electron microscopy (SEM), energy dispersive X-ray spectroscopy (EDX), and X-ray diffraction (XRD). The Rietveld analysis of the XRD data illustrates that both CuNi films have a nanosized crystallite size. Contact angle measurements reveal that the porous CuNi film exhibits remarkable superhydrophobic behavior, and fully dense CuNi film shows hydrophilicity. This is predominately ascribed to the surface roughness of the two films. The HER activity of the two prepared CuNi films were investigated in 1 M KOH solution at room temperature by polarization measurements and electrochemical impedance spectroscopy (EIS) technique. Porous CuNi exhibits an enhanced catalysis for HER with respect to fully dense CuNi. The HER kinetics for porous film is processed by the Volmer–Heyrovsky reaction, whereas the fully dense counterpart is Volmer-limited. This study presents a clear comparison of HER behavior between porous and fully dense CuNi films.

## 1. Introduction

Creating porosity of metals is nowadays considered as a promising strategy towards the development of materials with improved surface-related properties, thus creating opportunities for advanced technological application [1]. Amongst the surface-related properties of metals that can be enhanced by pore creating, hydrogen evolution reaction currently receives considerable attention from both scientific and applied perspectives [2,3,4]. Of the studies that exist, the focus mainly lies in understanding the kinetics of hydrogen evolution reaction (HER) from a fundamental perspective, and decreasing the overpotential to achieve high activities [5,6,7]. The porosity-related effect to electrocatalytic activity has been intensively studied by controlling the pore size or architecture [8,9,10,11]. Porous metals possess a high specific surface area and intrinsic reaction mechanism compared to their fully dense counterparts. Many attempts have been made to explain the enhancement of the electrocatalytic activity, including a shortened average distance between the reactants, increasing crystal porosity to increase active sites and the inner electrode surface, and boosted dynamics of the reactant in a confined space [12,13,14,15].

Electrodeposition (ED) processes constitute a low-cost and versatile technology for the production of porous metals and metallic alloys [16,17,18]. By applying either a current or potential signal, porous films with pore sizes ranging from meso-size to micron-size can be grown onto conductive substrates [19]. Key aspect that determines pore size encompasses template selection, electrolyte formulation, and film growth rate [20]. The growth of metal is always guided by the template and porous structure appears after the template is removed. For example, by taking advantage of hydrogen co-evolution as a source of porosity, metallic foam with micro-sized pores and dendritic pore walls can be prepared by ED [21]. In the case of a mesoporous structure, metallic films with tunable pore architectures can be electrodeposited in the presence of a triblock copolymer surfactant P123 [22]. Importantly, the ED of porous materials can operate near room temperature from aqueous electrolytes and can be used to grow a patterned porous structure through complex 3D masks [23,24].

Among metallic alloys, the Cu-Ni system has sparked the attention of researchers owning to its good electrocatalytic activities towards HER and its key roles in various electrochemical processes [25,26,27]. Typically, bimetallic CuNi materials show enhanced electrocatalytic activity and selectivity compared to monometallic Ni [28,29]. Porous cathodes exhibit, in comparison to fully dense electrodes of identical composition, lower onset potential and higher current density in HER [30]. It is a good candidate to explore the porous effect over HER performance because it shows total miscibility over the complete range of composition and can be electrodeposited from a water-based electrolyte [31]. Moreover, this system exhibits interesting functional properties such as resistance to corrosion, high tensile strength, and good wear resistance depending on the composition. Recently, our group demonstrated the template-assisted electrodeposition of microporous and mesoporous CuNi films, in which the sample with the least Ni content shows the highest specific activity [21,32]. This is due to the surface composition and spatial arrangement exerting a remarkable influence on the hydrogen adsorption and desorption. Protsenko and coworkers demonstrated that the surface inhomogeneity of CuNi alloy will lead to unexpected HER behaviors and it is revealed that the structure and morphology are the reasons for improving the hydrogen evolution performance of the anodic-treated Ni-Cu alloy [33,34]. Despite the numerous studies performed regarding the beneficial effect of porous structure on HER performance, the reported data are, in some cases, inconsistent across different investigations.

This work is aimed at providing a more comprehensive framework by a clear comparison between porous and fully dense CuNi films. In this context, porous CuNi films were electrodeposited from acidic electrolytes to ensure the hydrogen bubble generation as a dynamic template to confine the nucleation events, whereas fully dense CuNi with the same composition were prepared from a different bath containing citric acid as the complexing agent and saccharine as grain refiner. The purpose of the present study is two-fold: the characterization of the two CuNi samples, and a direct comparison between the properties of porous and fully dense CuNi with the same composition. The morphology, surface roughness, wettability, microstructure, and electrocatalytic properties are systematically investigated, and key relationships between these properties outlined.

## 2. Materials and Methods

### 2.1. Synthesis of CuNi Films

Analytical grade reagents and ultrapure water were used to prepare the electrolyte. All electrodeposition experiments were carried out in a 3-electrode cell connected to electrochemical workstation (CHI 760E, Shanghai Chenhua Instrument Co., Ltd, Shanghai, China). The working electrode was a pure copper plate with an active area of 1.5 cm^2^. Prior to deposition, the copper plate was well-polished with 1000, 2000, and 3000-grit SiC papers consecutively to avoid any deposition nonuniformity and impurity induced by the cathode surface topography. After that, the copper surface was degreased with ethanol followed by sulphuric acid and water to remove any oxide residues presented on the surface. A platinum plate was used as counter-electrode. In addition, a double junction Ag|AgCl (*E* = + 0.210 V versus standard hydrogen electrode (SHE)) was used as reference electrode with 3 M KCl inner solution and an interchangeable outer solution was utilized as the reference electrode. CuNi porous film was electrodeposited from an electrolyte solution containing 131.43 g/L NiSO_4_·6H_2_O, 8.15 mL/L H_2_SO_4_, 8.60 mL/L HCl, and 2.50 g/L CuSO_4_·5H_2_O. The electrodeposition was performed galvanostatically at *j* = −0.67 A/cm^2^ at 25 °C and the deposition time was 100 s. As for fully dense CuNi film, the electrolyte contained 184 g/L NiSO_4_·6H_2_O, 11.65 g/L CuSO_4_·5H_2_O, 62.2 g/L C_6_H_8_O_7_·H_2_O (citric acid monohydrate), 0.2 g/L NaCl_2_H_25_SO_4_ (sodium lauryl sulphate), and 0.5 g/L C_7_H_5_NO_3_S (saccharin). The bath pH was brought to 4.5 by 10 M NaOH and the plating temperature was maintained at 30 °C. CuNi fully dense film was obtained galvanostatically by applying a constant current density of −0.03 A/cm^2^ for 1000 s. Before each deposition, the electrolyte was de-aerated with nitrogen gas (N_2_) flow and a blanket of N_2_ was kept on top of the solution during CuNi growth.

### 2.2. Characterization

Scanning electron microscopy (SEM, Carl Zeiss Jena, Oberkochen, Germany) images were studied on a Merlin Zeiss microscope operated at 3 kV. The chemical composition of the porous CuNi film was determined by energy-dispersive X-ray spectroscopy (EDX, Carl Zeiss Jena, Oberkochen, Germany) at 10, 12, 15, and 18 kV, and 15 kV for fully dense CuNi film. X-ray diffraction (XRD, Panalytical B. V., Almelo, The Netherlands) patterns of the CuNi films were recorded with a Philips X’Pert diffractometer in the 40–56° 2*θ*-range (step size = 0.03°, step time = 2 s) using Cu Kα radiation (λ = 0.15257 nm). Average crystallite size and phase percentages were estimated using the Rietveld full-pattern fitting procedure (GSAS software, EXPGUI, B.H.ToBy, Gaithersburg, USA version). 3D imaging software (VK-H1XAC, VK-150K, Keyence, Osaka, Japan version) was used to measure the surface roughness. LAUDA Scientific GmbH’s LSA-100 is a surface analyzer (Lauda-Königshofen, Germany) which was used to determine the contact angles for the porous surfaces and the fully dense surfaces. The 2 μL water droplets were utilized for the measurements of static contact angles at room temperature. The values reported are average of three measurements made on different areas of the sample surface.

### 2.3. Electrocatalytic Activity towards HER

The electrochemical activity of the two CuNi films towards HER was measured in the same 3-electrode system used for deposition. The CuNi films deposited on pure copper substrate were used as the working electrode. Polarization curves were recorded in a de-aerated 1 M KOH solution by cycling the potential between −0.3 V and 0.1 V at 50 mV s^−1^. The Tafel slope was recorded at a scan rate of 5 mV s^−1^ and calculated by the following equation, *ƞ* = *b* × *logj* + *a*, where *j* was obtained with 100% iR correction, the value of iR from electrochemical workstation CHI760E by using open circuit potential, η is the overpotential, and b is the Tafel slope. The electrochemical active surface area (ECSA) is a vital factor to determine the electrochemical activity. To study the ECSA of as-prepared catalysts, we used CV measurements by different scan rates in the non-faradaic potential range vs SHE. The current density difference (Δ*J* = *J*_a_ − *J*_c_) at multiple scan rates was used to estimate double layer capacitance (C_dl_), where the slope is two times C_dl_ (*J*_a_ and *J*_c_ are the current density of anode and cathode, respectively). The C_dl_ can be obtained by the equation C_dl_ = $\frac{dQ}{dU}$ = $\frac{dQ/dt}{dU/dt}$ = $\frac{dJ}{dv}$, where *Q* is the charge associated with movement of electrolyte ions and adsorption/desorption at the electrode–electrolyte interface, *U* is the applied potential, *J* is the corresponding current density, and *v* is the scan rate. Electrochemical impedance spectroscopy (EIS) was performed using an amplitude signal of 5 mV from 10^5^ Hz to 10^−2^ Hz. Chronopotentiometry (CP) measurements and 50 cycles of linear sweep voltammetry (LSV) were run in order to assess material stability. The temperature of the cell was set at 25 °C.

## 3. Results

### 3.1. Morphology and Structure of CuNi Films

**Figure 1:**
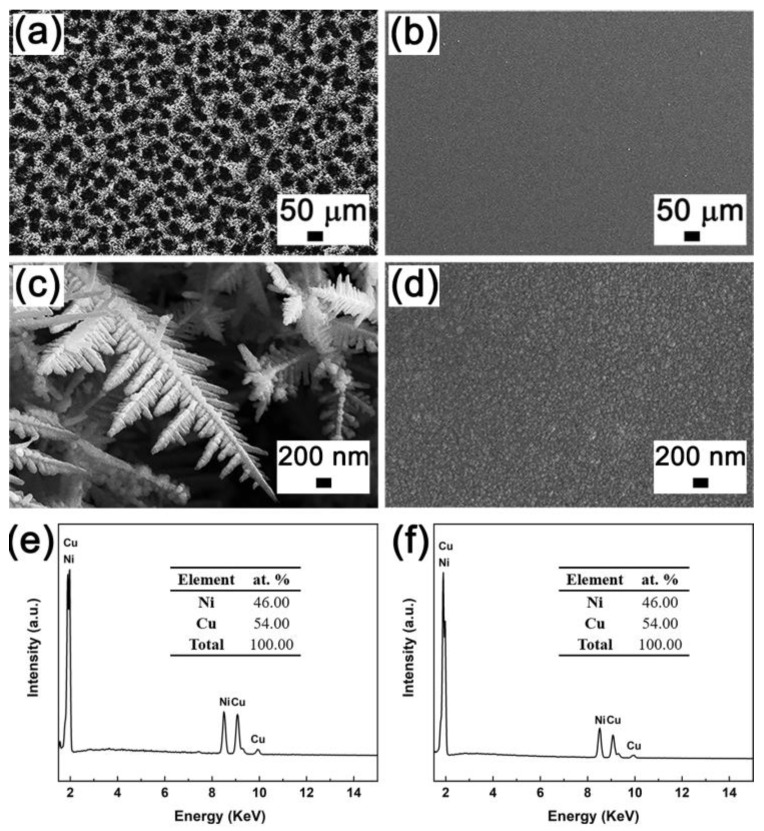
SEM (**a**,**b**) and zoomed SEM (**c**,**d**) images, and the corresponding EDX spectra (**e**,**f**) of porous (**a**,**c**,**e**) and fully dense (**b**,**d**,**f**) CuNi films.

Porous and fully dense CuNi films were successfully fabricated on pure copper substrates by electrodeposition. The deposition of porous CuNi was carried out at relative negative current densities and in acidic conditions to ensure the generation of vigorous hydrogen bubbles. The Cu and Ni discharge mostly at the interstices left by the bubbles, creating foam-like porous morphology. Moreover, the applied current density is rather high, and the electrodeposition process is in turn mass-transfer-controlled, and dendritic growth is favored. The growth morphology is with macro-sized pores and nanodendritic pore walls. The bath employed for the fabrication of fully dense CuNi is sulphate-based and contains saccharine as a grain refiner. The typical dendrite growth is suppressed, leaving silvery-bright appearance. Saccharine has long been used to grow smooth and almost featureless deposits irrespective of the applied current density [35,36,37]. It is suggested that the Cu reduction is diffusion controlled and it is mainly responsible for the formation of dendritic morphology. The addition of saccharine could block the diffusion of Cu ions and thus hinder Cu deposition. The SEM images of the two CuNi films are shown in Figure 1a,b. It is clearly found that the as-prepared porous CuNi film exhibit 3D interconnected porous structure with pore size ranging from 20–60 nm. A zoomed image depicts that the pore wall is made of loosely packed nanodendrites (Figure 1c). Fully dense CuNi demonstrates smooth and nearly featureless morphology (Figure 1d), indicating a decrease in surface roughness. Representative EDX spectra of the two films are shown in Figure 1e,f, in which Cu and Ni elements, with similar composition of Cu_54_Ni_46_, are detected.

**Figure 2:**
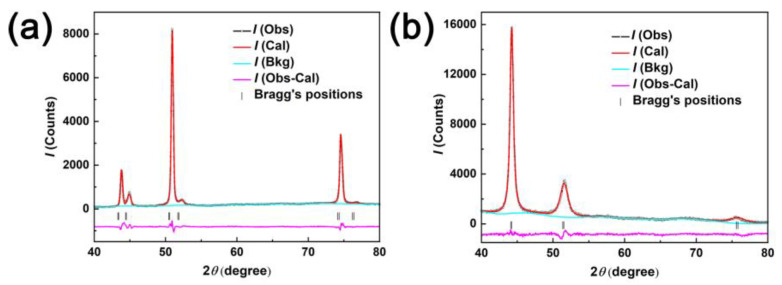
XRD patterns of porous (**a**) and fully dense (**b**) CuNi films in the 40°−80° 2θ range. I(Cal) and I(Bkg) stands for experimental powder diffraction, Rietveld-refined, and instrumental background data. The black vertical lines represent the Bragg reflection peaks and the I(Obs-Cal) is the differential intensity between I(Obs) and I(Cal).

**Figure 3:**
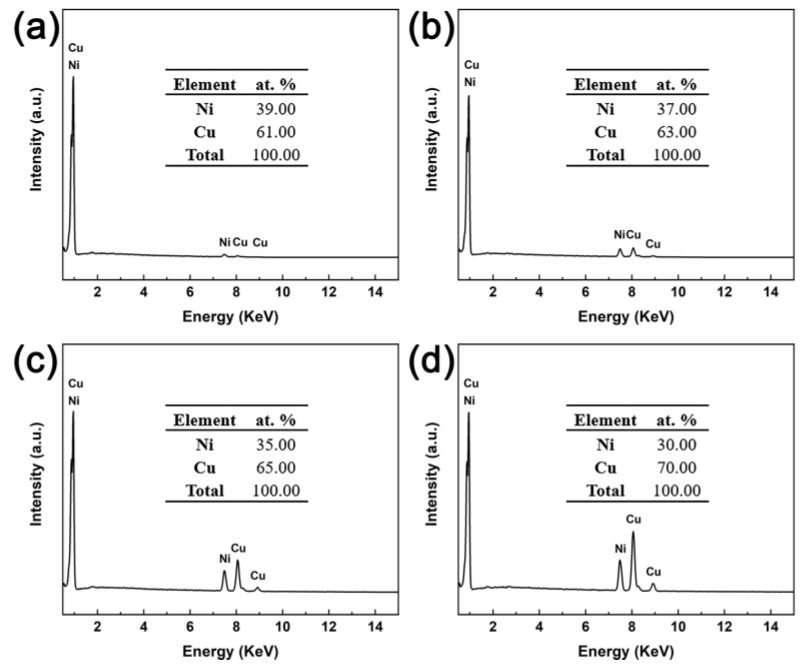
EDX spectra of porous CuNi film carried out under different accelerating voltages: (**a**) 10 kV, (**b**) 12 kV, (**c**) 15 kV, (**d**) 18 kV.

The structural properties of the porous and fully dense CuNi films were studied by XRD. The calculated and the experimental diffraction profiles together with the difference curves obtained after final refinement are shown in Figure 2. For fully dense CuNi, broad diffraction peaks can be detected, which match those of the face-centered cubic (fcc) Cu-Ni phase. Citrate plays a key role in reducing the large difference of the standard reduction potential of Cu (+0.34 V) and Ni (−0.25 V), thus allowing for their co-deposition [38]. However, six diffraction peaks corresponding to Cu (111), Ni (111), Cu (200), Ni (200), Cu (220), and Ni (220) reflections of the fcc structure are observed. The significant peak splitting suggests the phase separation and hence the formation of CuNi film with two phases. Moreover, in Figure 2, the calculated intensities and experimental data are shown by black point lines and red lines, respectively. The bottom blue line represents the difference between experimental and calculated data and the vertical lines below the diffraction pattern indicates the position of possible Bragg reflections of two CuNi films. It could be seen that the profiles for observed and calculated ones are matching with each other. The reliability factors (R-factors) R_p_, R_wp_, R_e_, and χ2 (goodness of fit (GOF)) obtained from refinement of all the two samples are listed in Table 1. The GOF values were sufficiently low, which indicates a good fit between the data calculated by the theoretical model and the observed patterns [39]. Furthermore, according to the analysis of XRD data from GSAS software (Table 1), the amount of Ni and Cu is estimated to 46 at% and 54 at% for fully dense CuNi, identical to the result of the EDX spectrum, while the porous CuNi film consists of pure Cu and pure Ni phases, whose contents are estimated to be 73 at% and 27 at%. This observation is contradictory to that of the EDX results. In the bath to prepare porous CuNi film, Ni and Cu discharge from uncomplexed Cu and Ni ions due to the lack of a complexing agent. Moreover, it is well-established that Cu^2+^ reduces much fast than Ni^2+^, leading to the variation in composition along the vertical direction to the electrode [40]. In addition, in the process of electrodeposition, the layer-growth habit also makes the components of the product vertically distributed [41]. Figure 3 is the EDX spectra of porous CuNi film carried out under different voltages. It is found that the Cu content increases together with the increasing accelerating voltage, suggesting a considerable range in chemical composition along the vertical direction. Nevertheless, the electrochemical performance of porous and fully dense CuNi films is still comparable, since it is a surface-related property, and the surface compositions of both samples to a certain depth are similar.

### 3.2. Surface Roughness

**Figure 4:**
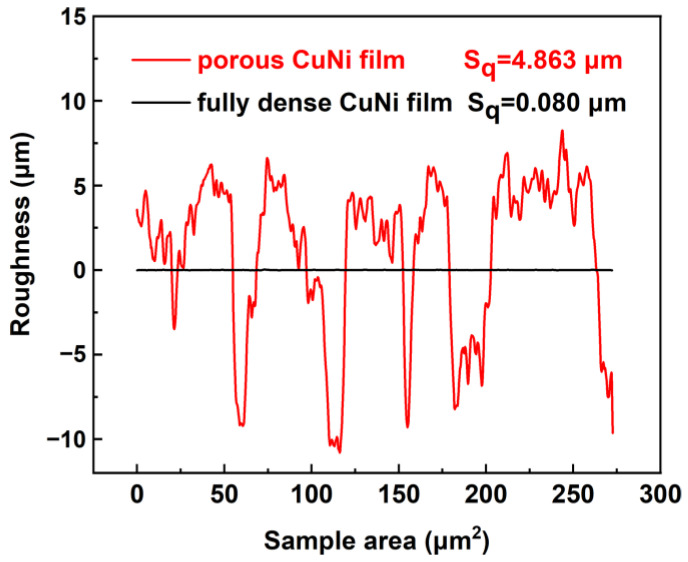
Roughness of porous and fully dense CuNi films.

**Figure 5:**
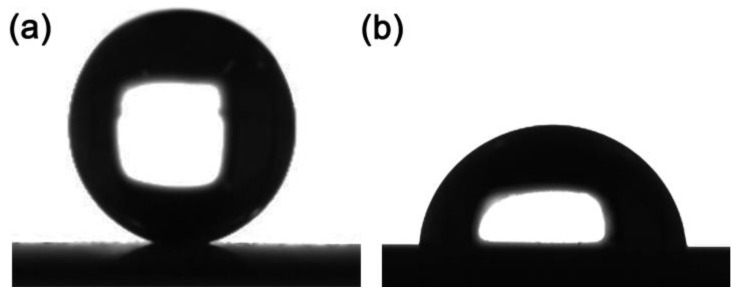
Contact angles of porous (**a**) and fully dense (**b**) CuNi films.

The surface roughness has a major impact on the quality and performance of the prepared two CuNi films. The root mean square roughness (S_q_) is 4.863 μm for porous CuNi and 0.080 μm for fully dense CuNi, as plotted in Figure 4. The Sq roughness parameter is a measure of the deviations in surface from the mean plane with the sampling area [42]. The roughness of porous CuNi is distributed as excessive undulations of around 5 to -10 μm in the vertical dimension. The fully dense CuNi is substantially smoother. Wettability of the two CuNi samples was evaluated using the sessile drop method. A 2 μL water droplet was deposited on the surface. Figure 5 shows that porous film exhibits a contact angle of around 158° and 84° for fully dense film. The 3D interconnected porous structure together with the dendritic walls results in the increase of the surface roughness, which will in turn affect the electrochemical performance.

### 3.3. Electrocatalytic Performance

**Figure 6:**
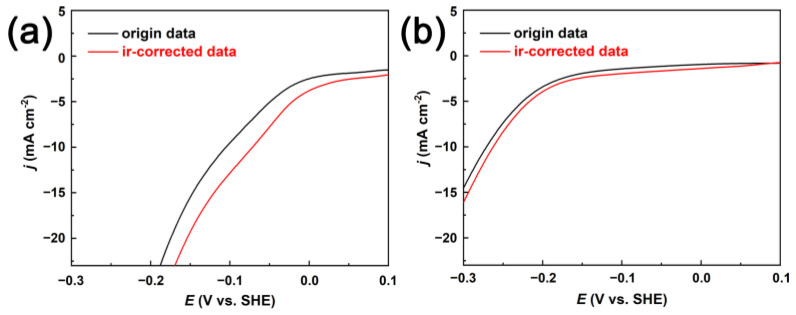
Polarization curves recorded in N_2_-saturated 1 M KOH electrolyte for porous (**a**) and fully dense (**b**) CuNi films. Scan rate: 50 mV s^−1^.

In order to investigate the electrocatalytic activity of the prepared two CuNi films, polarization measurements were carried out, where the current density is normalized by the projected area (geometrical area). As shown in Figure 6, the curves were recorded in 1 M N_2_-purged KOH solution at a sweeping rate of 50 mVs^−1^. Note that the curves are corrected with respect to the SHE and for the iR drop. The CuNi porous film exhibits superior HER activity with a relatively low overpotential of 72 mV at a current density of 10 mA cm^−2^. This value is comparable with that of the reported two-dimensional metal–organic-framework-derived porous nanoarrays [43]. However, the fully dense CuNi suffered a dramatic decrease, and the overpotential at 10 mA cm^−2^ is 263 mV. The enhanced HER activity can be attributed to the fast diffusion of the electrolyte into the electrode and abundant active sites provided by the micron-sized pores and highly interconnected nano dendritic pore walls.

**Figure 7:**
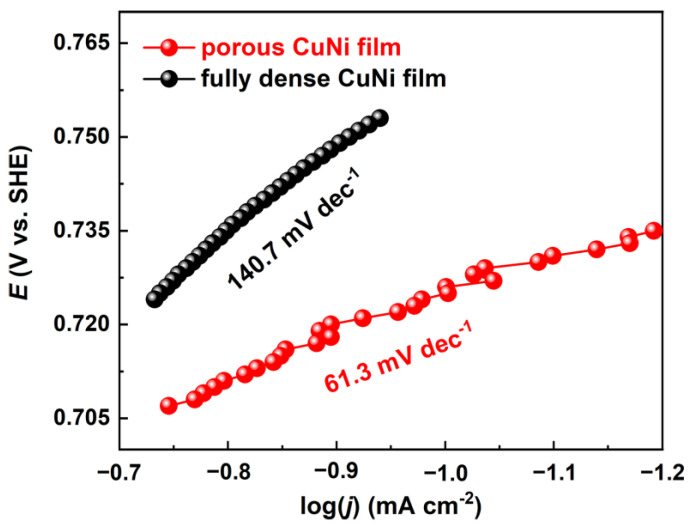
Tafel slopes of porous and fully dense CuNi films obtained from the HER polarization curves. Scan rate: 5 mV s^−1^.

In order to deeply understand the HER kinetics of the as-prepared films, the corresponding Tafel plots were derived from the polarization curves. In alkaline media, the HER takes place on the electrocatalyst surface via multiple electrochemical steps [44]: (1) The water molecule attacks the metal surface and then dissociates, leaving an adsorbed hydrogen atom and a hydroxyl group (H_2_O + e^−^ + M ⇌ M-H + OH^−^). (2) The electrochemical desorption process (M-H + H_2_O + e^−^ ⇌ H_2_ + OH^−^ + M, Heyrovsky reaction), or chemical desorption process (2 M-H ⇌ H_2_ + 2 M, Tafel reaction): Here, M refers to the vacant site of the electrocatalyst surface. As shown in Figure 7, porous CuNi film yields a Tafel slope of 61.3 mV dec^−1^, which is in between the theorical values of Volmer (120 mV dec^−1^) and Heyrovsky (40 mV dec^−1^) cases, indicating that the HER is proceeding through a Volmer–Heyrovsky reaction. However, significant change was observed in the slope of fully dense CuNi, the value of which is 140.7 mV dec^−1^. The change indicates that the Volmer step is the rate determine step (RDS) for fully dense CuNi [45]. The difference in HER kinetics can be ascribed to the surface structure. Low surface area leads to low surface hydrogen coverage for fully dense CuNi, as the adsorbed hydrogen would be consumed quickly by the fast Heyrovsky step. As for porous CuNi, the surface hydrogen coverage is boosted by the large surface area, increasing the Tafel slope from 140.7 mV dec^−1^ to 61.3 mV dec^−1^.

**Figure 8:**
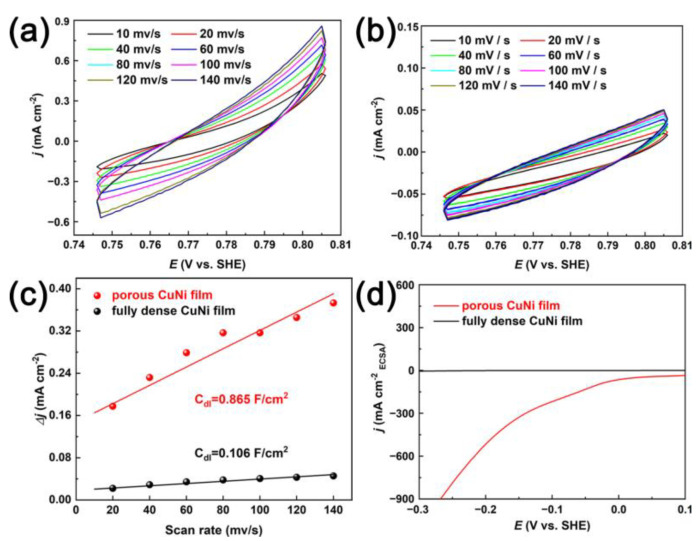
CVs of porous (**a**) and fully dense (**b**) CuNi films at various scan rates (10–140 mV/s) in the potential region of 0.747–0.805 V vs. SHE. (**c**) Capacitive current densities at 0.776 V (vs. SHE) as a function of scan rate for porous and fully dense CuNi films. (**d**) Polarization curves of the two CuNi films calculated by ECSA.

Complementary proof for the HER mechanism was obtained from the assessment of ECSA determined by C_dl_. Figure 8 depicts the CV curves collected in the region of 0.747–0.805 V (vs. SHE), where the current response should only be because of the charging of the double layer. The C_dl_ was calculated from the slope of linear fit of the current density at 0.776 V against scan rates (Figure 8c). The C_dl_ of the porous CuNi film (0.865 F/cm^2^) catalyst is much larger than that of the fully dense counterpart (0.106 F/cm^2^). Subsequently, the polarization curves of porous and fully dense CuNi film were normalized using ECSA instead of geometric area, as shown in Figure 8d. The trend is pretty much similar to Figure 6, in which porous CuNi film had a lower initial overpotential than that of fully dense CuNi film. In summary, the larger surface coverage of active sites in porous CuNi film promotes the electrocatalytic reaction rate and hydrogen production.

**Figure 9:**
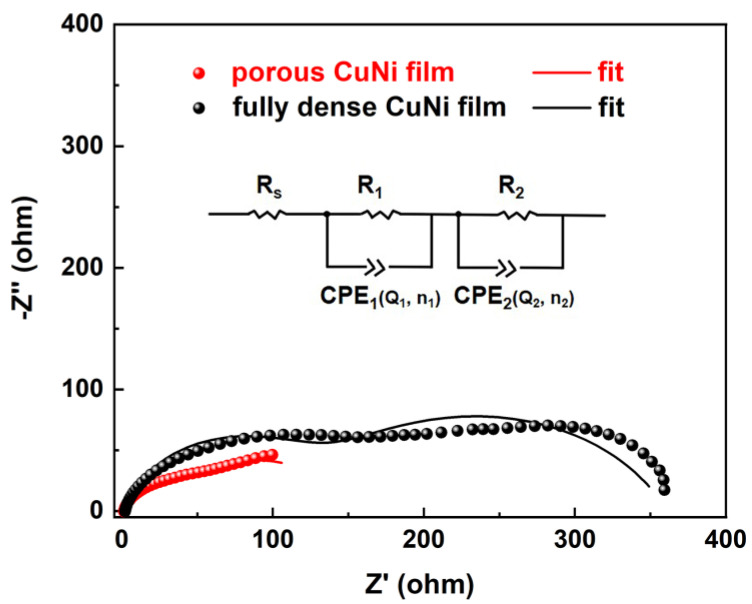
EIS Nyquist plots of porous and fully dense CuNi films in 1 M KOH solution. The inset is the equivalent circuit. R_s_ represents the electrolyte resistance, R_1_ is the charge transfer resistance, R_2_ is the diffusion transfer resistance, and CPE is the conventional double-layer capacitance to account for non-uniform diffusion.

Moreover, EIS was conducted to confirm the HER kinetics on porous and fully dense CuNi films. The corresponding Nyquist plots are shown in Figure 9, of which two semicircles can be detected. The EIS Nyquist plot is also presented in terms of the equivalent circuit model (inset in Figure 9). A similar electrolyte resistance (R_s_) was calculated, which was ascribed to the ion migration behavior in the electrolyte. In this case, the constant phase element (CPE) is also considered, due to the fact that the atomic scale inhomogeneities of the electrodes surface exhibits a fractal character [46]. Therefore, the electric double layer capacitance is roughly determined from CPE, of which the impedance can be calculated from: Z(CPE) = Y_0_^−1^(jw)^−n^. Y_0_ is a parameter related to the double layer capacitance, while j is the imaginary unit (j =$\;\sqrt{-1}$) and w is the angular frequency (w = 2πf, f being the frequency), and n is a dimensionless parameter ranging between 0 and 1 at a solid electrode/solution interface. When the equation is used to describe an ideal capacitor, the constant Y_0_ = C (the capacitance) and n = 1 [47]. CPE_1_ and R_1_ were used to describe non-faradaic processes in the impedance frequency area, and CPE_2_ and R_2_ were used to describe the low frequency region of the charge-transfer process [48].

According to the corresponding equivalent circuits, the calculated values (derived from ZView software, 3.1, Ziffnet, San Francisco, USA version) of these parameters are listed in Table 2. As shown in the table, the charge-transfer resistance (R_2_) of the CuNi films were larger than the diffusion resistance (R_1_), indicating that charge transfer is the controlling step in the HER process [49]. In addition, R_1_ of porous CuNi film was smaller than that of fully dense CuNi film, which further indicts that the enhanced HER activity of porous CuNi film can be attributed to the fast diffusion of the electrolyte into the electrode. The calculated R_2_ for porous CuNi (104.90 Ω cm^2^) is much smaller compared with that of the fully dense CuNi (237.60 Ω cm^2^), indicating a lower resistivity against charge transfer on the interface between the film and electrolyte. Consequently, the electron transfer of porous CuNi, which correlates with the HER activity, is enhanced [50,51]. In addition, the higher value of CPE_2_ for porous CuNi indicates a larger available surface active site, as it is proportional to surface area [52].

**Figure 10:**
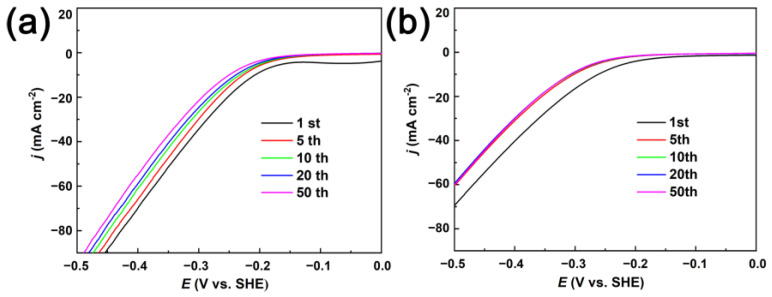
LSV curves of porous (**a**) and fully dense (**b**) CuNi films in N_2_-saturated 1 M KOH solution.

**Figure 11:**
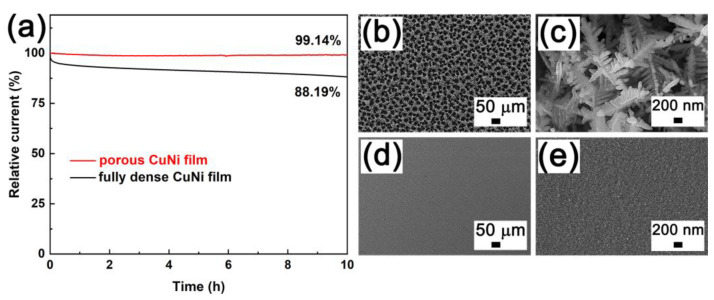
Chronopotentiometry response of porous CuNi film and fully dense CuNi film (**a**). SEM images of porous (**b**,**c**) and fully dense (**d**,**e**) CuNi films after 10 h durability tests.

Alkaline electrolysis is one of the most powerful technologies to produce hydrogen energy, where the electrocatalysts play an important role. However, in electrocatalysis processes, catalysts may suffer from loss of catalytic activity [47]. Therefore, the long-term durability of porous and fully dense CuNi films were investigated by 50 cycles of LSV scanning in 1 M KOH electrolyte within the potential range of −0.5 V to 0 V. As shown in Figure 10, it is seen that the porous and fully dense films demonstrate excellent time stability and superior hydrogen evolution activity in concentrated alkaline solution. However, note that with the increase of the number of cycles, the potential onset of porous CuNi film was in the trend of moving to the negative value under the same current density. This is primarily due to the fact that the release of hydrogen aggravates the charge transfer at the metal to solution interface. On the other hand, HER current density of porous CuNi has the highest value at the first cycle, and decrease gradually during the subsequent cycles. However, after the fifth cycle for fully dense CuNi, the current densities are maintained and stable LSV curves are detected. This can be interpreted to be the reduced surface coverage with Volmer-limited reaction for fully dense CuNi. The adsorbed hydrogen would be consumed quickly by the relatively fast Heyrovsky and/or Tafel step (s). Thus, the hydrogen evolution requires a shorter time to reach the equilibrium state of surface tension and buoyancy force. In addition, the long-term stability was measured by the chronopotentiometry method at a fixed current density of 10 mA cm^−2^ for 10 h. Figure 11a shows the stability of porous CuNi film, with 99.14% retention of the initial current density, which is higher than that achieved with fully dense film (88.19%). It is observed that the porous CuNi film exhibits excellent electrochemical activity with outstanding stability. Furthermore, the SEM image of the sample after a 12 h reaction provided in Figure 11b–e). The morphologies demonstrated no pronounced changes indicating the high durability of the prepared CuNi films.

## 4. Conclusions

To summarize, ED is a powerful method to prepare metallic films with varying spatial arrangement, composition, and morphology. From an aqueous-based solution we successfully synthesized both fully dense and porous CuNi films with comparable composition. The prepared two CuNi films were used as cathodes for HER. A detailed structural characterization by XRD reveals that both of the films exhibit a nanocrystalline nature (with crystallite sizes around 50 nm for porous CuNi, and 30 for fully dense CuNi). The CuNi porous film exhibits superior HER activity with a relatively low overpotential of 72 mV at current density of 10 mA cm^−2^. A comparison of HER kinetics in 1 M KOH indicates that the higher HER activity of porous CuNi film is due to its higher ECSA. It is shown that the porosity clearly improves electrocatalytic properties towards HER.

## Figures and Tables

**Table 1:** Composition of porous and fully dense CuNi film, as determined from XRD analyses.

**Porous** **CuNi**	**Ni phase** d = 2.015 a = b = c = 3.53181 at% Ni = 27% average crystallite size 60 nm	**Cu phase** d = 2.064 a = b = c = 3.61597 at% Cu = 73% average crystallite size 55 nm	R_p_ (%) = 4.03 R_wp_ (%) = 5.71 R_e_ (%) = 5.35 GOF = 1.07
**Fully dense CuNi**	d = 2.044 a = b = c = 3.55516 at% Ni = 46% at% Cu = 54% average crystallite size 32 nm		R_p_ (%) = 2.10 R_wp_ (%) = 2.78 R_e_ (%) = 1.70 GOF = 1.63

**Table 2:** Best-fit estimates of electric equivalent circuit parameters obtained from the impedance measurements of the porous and fully dense CuNi films in 1 mol/L KOH at 25 °C.

Sample	Porous CuNi Film	Fully Dense CuNi Film
R_s_ (Ω cm^2^)	1.50	2.06
Q_1_ (S^n^ Ω^−1^ cm^−2^)	1.58 × 10^−3^	5.04 × 10^−5^
n_1_	0.90	0.87
R_1_ (Ω cm^2^)	39.75	121.50
Q_2_ (S^n^ Ω^−1^ cm^−2^)	1.03 × 10^−2^	1.20 × 10^−3^
n_2_	0.82	0.71
R_2_ (Ω cm^2^)	104.90	237.60

## Data Availability

The data presented in this study are available on request from the corresponding author.
